# Influence of body position during Heimlich maneuver to relieve supralaryngeal obstruction: a manikin study

**DOI:** 10.1002/ams2.297

**Published:** 2017-07-17

**Authors:** Michitaro Ichikawa, So Oishi, Katsunori Mochizuki, Kenichi Nitta, Kazufumi Okamoto, Hiroshi Imamura

**Affiliations:** ^1^ Department of Emergency and Critical Care Medicine Shinshu University School of Medicine Matsumoto Japan

**Keywords:** Adult, airway obstruction, child, Heimlich maneuver, prone position

## Abstract

**Aim:**

To study the most effective body position for Heimlich maneuver.

**Methods:**

A choking simulation manikin was connected to a laryngeal model of a child or an adult, and a differential pressure transducer recorded the airway pressure and waveform during the maneuver. A konjac jelly was placed on the larynx to mimic complete supralaryngeal obstruction. The maneuver (five successive compressions) was carried out six times each in standing, prone, and supine positions. For cases of children, we added a supine position with a pillow under the back.

**Results:**

In the adult model, airway obstruction was more frequently relieved in the supine and prone positions than in the standing position (*P* < 0.001). In the child model, airway obstruction was more frequently relieved in the supine position, with a pillow, and in the prone position, than in the standing position (*P* < 0.001). Without relief, successive Heimlich maneuvers made the airway pressure increasingly negative (adult, from −21.9 ± 6.5 cmH_2_O to −31.5 ± 9.1 cmH_2_O in the standing position [*P* < 0.001]; child, from −15.0 ± 9.5 cmH_2_O to −30.0 ± 9.2 cmH_2_O in the standing position [*P* < 0.001] and from −35.0 ± 17.4 cmH_2_O to −47.3 ± 25.1 cmH_2_O in the supine position without a pillow [*P* = 0.002]).

**Conclusions:**

The Heimlich maneuver was more effective in the supine and prone positions. In children, the prone position may be most effective. Successive Heimlich maneuvers may be harmful when the airway is not relieved after the first compression.

## Introduction

Choking on food is one of the most frequent causes of accidental death in children and aged people.^1^, ^2^ The Heimlich maneuver was first reported as a first aid measure to prevent choking in 1974, and in 1975, 162 patients were saved by this maneuver.^3^ The basis of this maneuver is the creation of an artificial cough by forcefully elevating the diaphragm and forcing air from the lungs.

Choking can occur in various ways, such as obstruction in the mouth and nose, oropharynx, supralarynx, and trachea. Because it is difficult to know the level of obstruction, except when it occurs in the mouth and nose, the effectiveness of the Heimlich maneuver in complete supralaryngeal obstruction and at body positions other than standing remains largely unknown. We hypothesized that the standing position is not always the best for the Heimlich maneuver. Here, we studied the effect of the Heimlich maneuver on the supralaryngeal obstruction in the standing position, compared to other positions (supine and prone position), using a manikin as a choking model. This is the first study to investigate the success rate of the Heimlich maneuver in three positions by recording airway pressure during the maneuver.

Semi‐solid foods pose the highest risk of choking:^4^ the US Food and Drug Administration and the Food Standards Agency (UK) have issued warnings of the dangers of choking on a jelly containing konjac.^5^, ^6^ Konjac jelly does not dissolve readily and its surface becomes smooth and slippery when placed in the mouth.^7^ Between 1995 and 2008, 17 people died from choking on konjac jelly.^8^ Thus, in this study, we chose to use a konjac jelly that could reproduce complete supralaryngeal obstruction.

## Methods

### Experimental system

**Figure 1:**
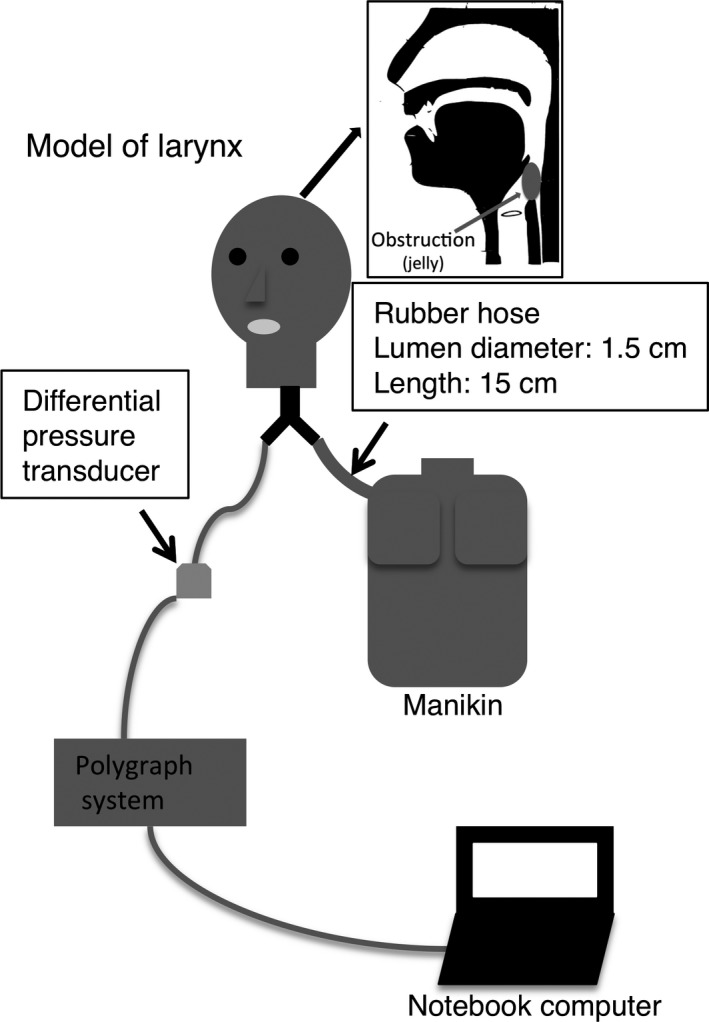
Experimental devices for studying the influence of body position during the Heimlich maneuver to relieve supralaryngeal obstruction. The obstruction (konjac jelly) was set on the larynx of a laryngeal model. The laryngeal model of a child or an adult was connected to both the manikin and the differential pressure transducer. The transducer was connected to the polygraph system. The polygraph system was connected to a notebook computer to record the waveform of airway pressure.

A laryngeal model of an adult (Laerdal Airway Management Trainer, head, skin, & airways ALS/AMT [25200]; Laerdal, Ampat, Singapore) and a child (Laerdal Pediatric Intubation Trainer, Pediatric Intubation Trainer Torso; Laerdal) were individually connected to a choking simulation manikin (Laerdal Choking Charlie; Laerdal) (Fig. 1). To measure airway pressure, a differential pressure transducer and a polygraph system were used. These were connected to a notebook computer running LabChart 7 version 7.2.2 software (https://www.adinstruments.com/products/labchart) (Fig. 1). An electronic spirometer (SP‐370COPD; Fukuda Denshi, Tokyo, Japan) was used to measure the expiratory volume of the manikin.

### Study protocol

Five emergency physicians with Immediate Cardiac Life Support certification participated in this study after giving written informed consent.

First, we measured the expiratory volume of the manikin produced by the Heimlich maneuver with no foreign body in the airway. Then, we placed konjac jelly, which is readily commercially available in Japan, of 4.3 × 3.0 × 3.0 cm dimensions, on the larynx of the manikin.

The Heimlich maneuver was performed by each of the participants on the manikin five times successively in one procedure set. Six sets of the procedure were performed in each of the standing, prone, and supine positions. For the child model in the supine position, an additional position, the supine position with a pillow placed under the back of the laryngeal model, was adopted.

During each of the maneuvers, in each position, we measured the expiratory volume of the manikin and recorded the waveform of the airway pressure. When the jelly was removed after a single procedure set (i.e., five compressions), the procedure was defined as an “opened case”, and when the jelly was not removed, it was defined as an “unopened case”.

The primary outcome of this study was the number of opened cases in each position. The secondary outcome was the change of airway pressure during the Heimlich maneuver.

### Setting of each position

#### Standing position

The manikin was set on a table vertically and the experimenter took up the position behind it, with his arms encircling the chest, and compressed the abdomen immediately above the umbilicus.

#### Supine position

The manikin was laid on its back on the floor. The experimenter sat astride the manikin body and compressed the abdomen immediately above the umbilicus.

#### Prone position

The manikin was laid with its face toward the floor and the experimenter placed himself over the manikin from behind, with his arms encircling the chest, and compressed the manikin's abdomen upwards, immediately above the umbilicus.

### Data collection and analysis

Data are shown as means ± standard deviation. Statistical analysis was carried out using spss 22.0 software (IBM SPSS, Chicago, IL, USA). The expiratory volume and airway pressure of each position were compared using one‐way anova. The χ^2^‐test was used for comparison of discrete variables. The Jonckheere*–*Terpstra test was used for comparison of trends of negative airway pressure in unopened cases. A *P*‐value of <0.05 was considered statistically significant.

## Results

### Expiratory volume produced by the Heimlich maneuver in the absence of a foreign body

**Figure 2:**
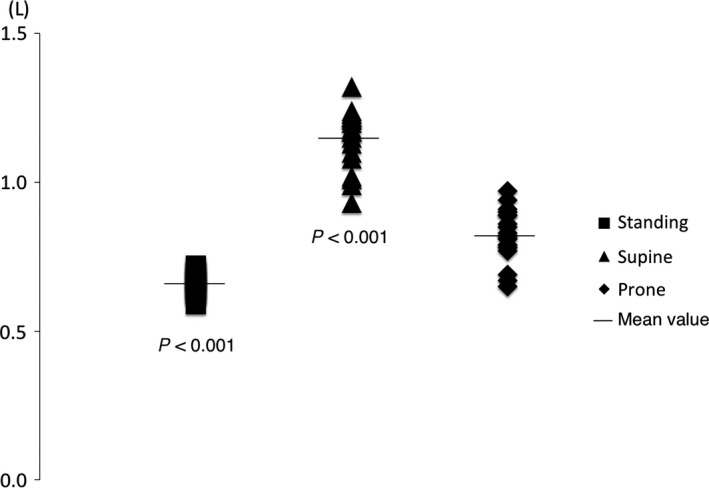
Expiratory volume produced from the manikin by the Heimlich maneuver in the absence of a foreign body. The expiratory volume was 0.66 ± 0.04 L, 1.15 ± 0.10 L, and 0.82 ± 0.09 L in standing, supine, and prone positions, respectively. These expiratory volumes were significantly different (*P* < 0.001). After Bonferroni correction, the expiratory volume was significantly greatest in the supine position, and significantly smallest in the standing position (*P* < 0.001).

The expiratory volume produced from the manikin by the Heimlich maneuver in the absence of a foreign body was significantly greatest in the supine position, and significantly smallest in the standing position (*P* < 0.001) (Fig. 2).

### Airway pressure in opened and unopened cases

**Figure 3:**
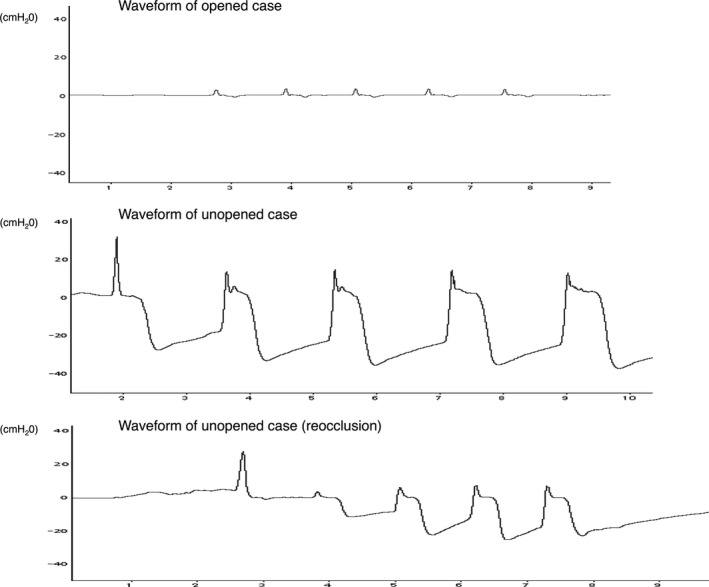
Waveform of the airway pressure in a manikin during the Heimlich maneuver. Opened airway (top): there was no obstruction of the larynx. Unopened airway (middle): the airway was not relieved during successive Heimlich maneuvers. The airway pressure showed a transient positive wave followed by a large negative pressure. The minimum airway pressure was −31.2 cmH_2_O, −37.4 cmH_2_O, −40.5 cmH_2_O, −40.4 cmH_2_O, and −40.5 cmH_2_O after the 1st, 2nd, 3rd, 4th, and 5th compression, respectively. Reocclusion case (bottom): the airway was first relieved and then obstructed again during the successive Heimlich maneuver. This waveform shows one such example (the airway was revealed after the 1st compression and obstructed again after the 2nd compression).

Figure 3 shows the airway pressure produced in the manikin. When there was no foreign body in the airway, there was little change in the airway pressure (top panel). In the case of an obstructed airway, the airway pressure showed a transient positive wave followed by a large negative wave. The minimum airway pressure of the negative became increasingly negative with each of the five successive compressions (middle panel). Thus, airway pressure can be used to determine whether the airway is obstructed.

The bottom panel of Figure 3 shows a reocclusion case. Once a foreign body was removed by the Heimlich maneuver, the airway pressure showed little change with the next compression, but after further compression, airway pressure became negative, in the same way as for an unopened case, indicating that the airway was obstructed again. We confirmed that the airway was relieved when the waveform of the airway pressure returned to baseline (0 cmH_2_O) after compression. Thus, opened cases were judged by observing the airway pressure.

### Effect of body position during the Heimlich maneuver in the adult laryngeal model

**Figure 4:**
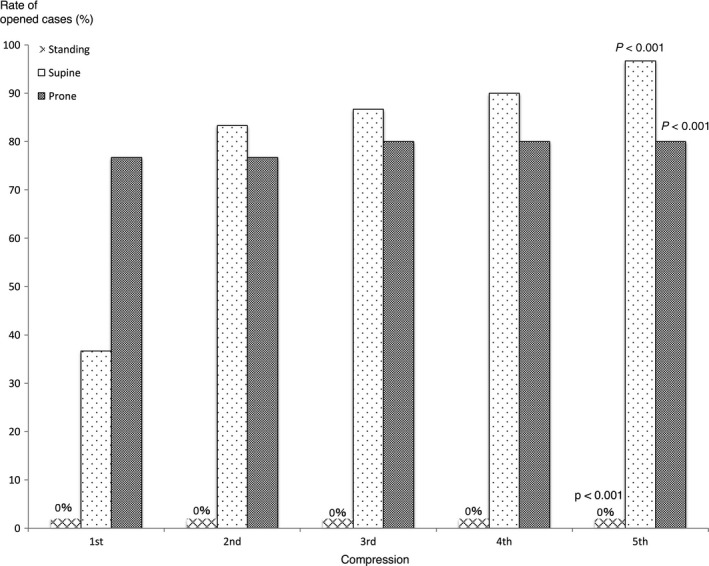
Rate of opened airway cases in each position of the Heimlich maneuver in adult models. After the 5th compression, the rate of opened cases was significantly lower in the standing position and significantly higher in the supine and prone positions (all *P* < 0.001).

Figure 4 shows the rate of airway obstruction relief in each position in the adult model. In the standing position, the airway could not be relieved at all. In the supine position, the rate of opened cases after five compressions was 97%. The single unopened case was a case of reocclusion. In the prone position, the rate of opened cases after five compressions was 80%. Both unopened cases were reocclusion cases. The rate of opened cases was significantly higher in the supine and prone positions than in the standing position.

**Figure 5:**
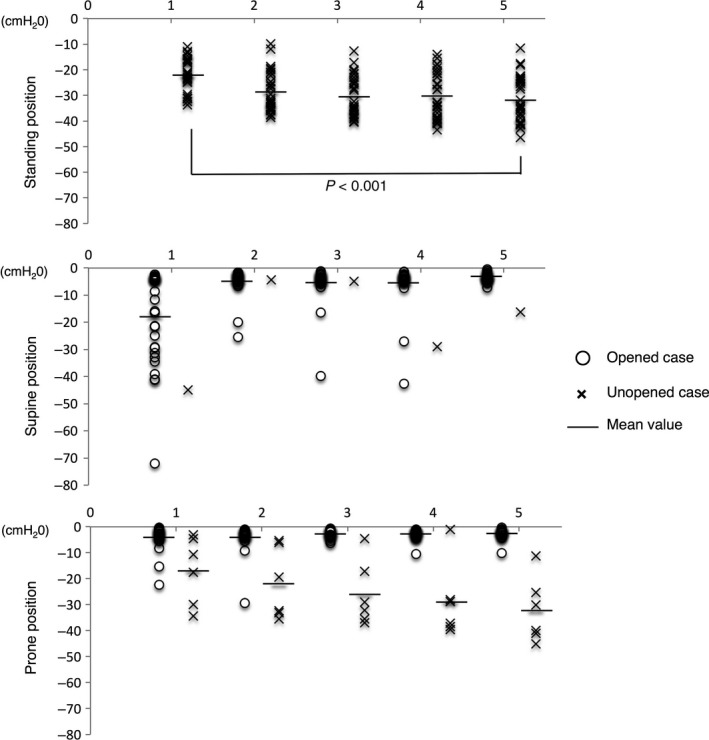
Minimum airway pressure after the Heimlich maneuver in adult models. In the standing position, the airway pressure significantly reduced with successive Heimlich maneuvers (*P* < 0.001). In the supine position, only one unopened case had reocclusion. The airway was relieved after the 2nd compression and obstructed again after the 4th compression. In the prone position, the unopened cases included two reocclusion cases. In one of these, the airway was relieved after the 1st compression and obstructed again after the 3rd compression. In the other case, the airway was relieved after the 1st compression and obstructed again after the 5th compression. Because the unopened cases included reocclusion cases, we did not use the Jonckheere*–*Terpstra test. The horizontal bar shows the mean value of airway pressure in opened cases and unopened cases, respectively.

Opened cases in both the supine and prone positions included cases classified as reopened cases after reocclusion. In the standing position, the airway pressure became negative after the Heimlich maneuver when the airway obstruction was not relieved. In addition, the airway pressure of unopened cases became significantly lower from −21.9 ± 6.5 cmH_2_O after the 1st compression to −31.5 ± 9.1 cmH_2_O after the 5th compression (Fig. 5, top panel).

In the unopened case in the supine position, the airway obstruction was first relieved after the 2nd compression, but was obstructed again after the 4th compression (Fig. 5, middle panel). In unopened cases in the prone position, the airway pressure became increasingly negative, but was not significantly different between compressions (Fig. 5, bottom panel).

### Effect of body position during the Heimlich maneuver in a child laryngeal model

**Figure 6:**
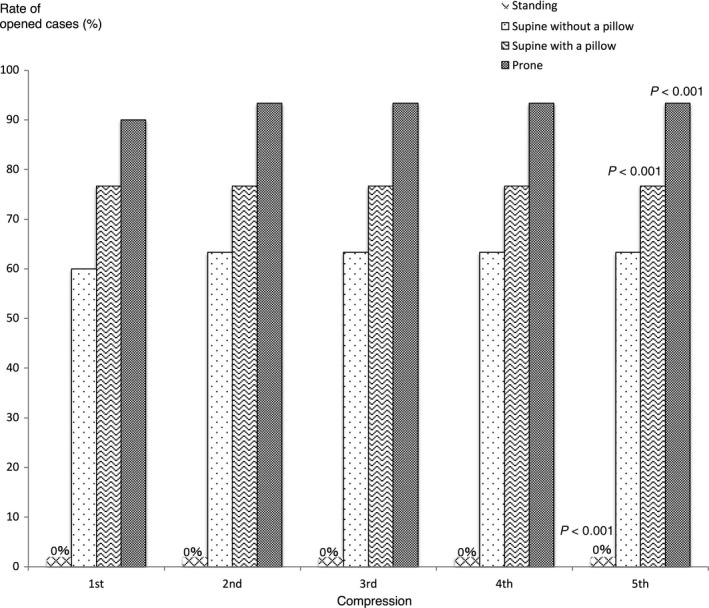
Rate of opened airway cases in each position of the Heimlich maneuver in a child model. After the 5th compression, the rate of opened cases was significantly lower in the standing position and significantly higher in the supine position with a pillow and in the prone position (*P* < 0.001).

Figure 6 shows the rate of airway obstruction relief in each position in the child model. In a standing position, the airway obstruction could not be relieved at all. In the supine position, the rate of opened cases was 63% after five compressions, whereas in the prone position, the rate of opened cases was 93% after five compressions. In the supine position with a pillow behind the back, the rate of opened cases was 77% after five compressions. The rate of opened cases was significantly higher in the supine position with a pillow and in the prone position than in the standing position, but there was no significant difference in the rate of opened cases between the supine position with and without a pillow.

**Figure 7:**
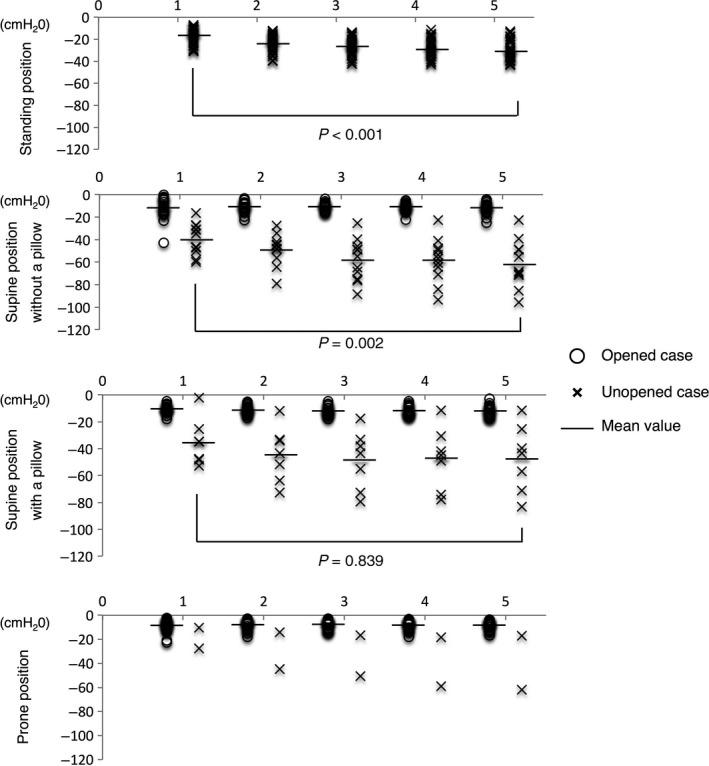
Minimum airway pressure after the Heimlich maneuver in a child model. Reocclusion cases were not observed in any of the unopened cases, in any of the positions in the child model. In the standing position, the airway pressure of 30 unopened cases was significantly reduced by successive Heimlich maneuvers (*P* < 0.001). In the supine position without a pillow, the airway pressure of 11 unopened cases was significantly reduced (*P* = 0.002). In the supine position with a pillow, the airway pressure of seven unopened cases did not show this trend to reduce (*P* = 0.839). In the prone position, there were only two unopened cases; therefore, we did not calculate the mean airway pressure. The horizontal bar shows the mean value of airway pressure in the opened cases and unopened cases, respectively.

As in the adult model, in the standing position, the airway pressure reduced increasingly with five compressions when the airway obstruction was not relieved. The airway pressure of unopened cases reduced significantly with each successive Heimlich maneuver (Fig. 7, first row). In unopened cases in the supine position without a pillow, the airway pressure also became significantly lower (Fig. 7, second row).

## Discussion

Our study showed that reocclusion may occur with successive Heimlich maneuvers and that the success rate of relieving the airway is higher in the prone and supine positions than in the standing position.

Previous studies reported that chest compression in several positions generates higher airway pressure than the Heimlich maneuver.^9^, ^10^, ^11^ The results of our manikin study are consistent with those of previous studies.

In unopened cases in both the child and adult models, the Heimlich maneuver generated a more negative airway pressure than in opened cases. This was because intrapulmonary air was ejected by the Heimlich maneuver, although new air could not be inhaled because the foreign body reoccluded the supralarynx when it had not been moved into the oral cavity. The Heimlich maneuver therefore poses a risk of lodging the foreign body more firmly in the larynx if it is not removed after the first compression. Continuing with repeated maneuvers will not only cause the airway pressure to become more negative, but will also increase the difficulty of removing the foreign body by reducing the remaining air that can be forced from the lungs. This risk is increased when performing the Heimlich maneuver in the standing position. In order to open the airway successfully, the Heimlich maneuver should be performed in a prone or supine position.

In the child model, the airway was relieved less frequently than in the adult model by the Heimlich maneuver performed in the supine position, but more frequently when in the prone position. The main reason is the narrowing of the airway by neck anteflexion of a child. A child's head is relatively large compared to the body, so that the neck is likely to be anteflexed in the supine position.^12^, ^13^, ^14^, ^15^ A pillow under a child's back was useful to avoid such neck anteflexion and increased the success rate of airway obstruction relief. In the prone position, gravity could also exert a positive effect, as the mouth faced toward the ground in the prone position. Because of the smaller laryngeal cavity, a foreign body may more easily fall into the oral cavity, due to gravity, in a child than in an adult.

In unopened cases, the foreign body could not be removed due to the increasing negative airway pressure and reocclusion caused by successive performance of the Heimlich maneuver. The current guidelines recommend that the Heimlich maneuver should be applied in rapid succession until a foreign body is relieved,^16^ and that it should be performed in the standing (or sitting) and supine positions. However, our findings suggest that successive Heimlich maneuvers may be hazardous and that it is better to perform the Heimlich maneuver in the supine and prone position in case of an adult and a child, respectively. If the prone position is not acceptable in a child, the supine position with a pillow behind the back may be a good alternative.

## Limitations

This study has several limitations. First, although the mechanism for elevating airway pressure is similar to that in a human, a manikin is not quite the same as a human; larynx models are different from real humans in flexibility and humidity. Nonetheless, the airway pressure in our manikin was similar to that of recently deceased adults and pigs, as reported in previous studies.^9^, ^10^, ^11^ Therefore, the results of our study may be applicable to human cases. Second, we used only konjac jelly as the obstruction material; therefore, we could not estimate whether other foreign bodies would create a similar larynx obstruction in the larynx. Third, although we used a child and an adult laryngeal model, the choking simulation manikin was that of an adult body. We did not estimate the difference in expiratory volume between a child and an adult. However, the expiratory volume of a child is smaller than that of an adult. If the airway obstruction cannot be relieved by the expiratory volume of an adult, it will probably not be relieved by the expiratory volume of a child. For this reason, we considered that it was not necessary to use a child choking model. Finally, because this was a manikin study, the adverse effects of compression in the prone and supine position were not evaluated.

## Conclusion

With a complete supralaryngeal obstruction, the Heimlich maneuver performed in the supine and prone positions may be more effective for adults and children, respectively, than that performed in the standing position in a choking simulation manikin. Successive Heimlich maneuvers may be harmful when the airway is not relieved after the first compression.

## Disclosure

Informed consent: All participants gave their written informed consent.

Conflict of interest: None declared.
